# Bilateral stellate neuroretinitis revealing a pheochromocytoma

**DOI:** 10.11604/pamj.2015.20.13.4794

**Published:** 2015-01-06

**Authors:** Samar Younes, Meriem Abdellaoui, Fadoua Zahir, Idriss Benatiya, Hicham Tahri

**Affiliations:** 1 Ophthalmology Service, University Hospital Center Hassan II, Fes, Morocco

**Keywords:** Stellate neuroretinitis, pheochromocytoma, optic disc edema, macular star

## Abstract

Neuroretinitis (NR) is an inflammatory disorder characterized by optic disc edema and subsequent formation of a macular star. We present a case of a 33 year old woman patient admitted for a progressive bilateral visual loss since two weeks. Fundus examination showed bilateral stellate neuroretinitis. Physical examination revealed a malignant hypertension of 210/150mmHg. Magnetic resonance imaging identified a left suprarenal mass, whereas urinary catecholamine level was abnormally high which supported a diagnosis of pheochromocytoma. The patient underwent a laparoscopic left suprarenal adrenalectomy after successful control of blood pressure. histopathologic examination confirmed the diagnosis of pheochromocytoma. Visual acuity was restored and the retinal alterations disappeared 7 months after surgery.

## Introduction

**Figure 1:**
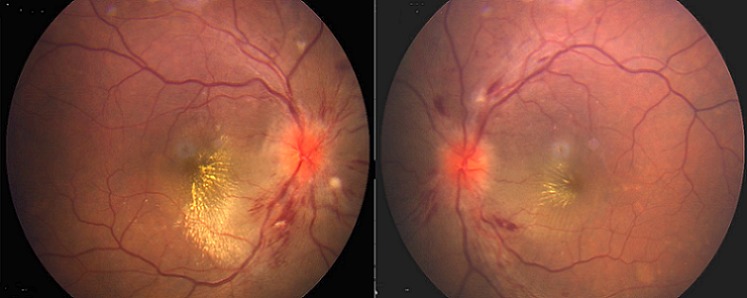
Fundus photograph showing bilateral optic disc edema, soft exudates, macular star, flame-shaped hemorrhages

Neuroretinitis (NR) is an inflammatory disorder characterized by optic disc edema and subsequent formation of a macular star [1] (Figure 1). The causes of this condition are numerous and are dominated by infectious and inflammatory etiologies in the young subjects and vascular etiologies in older patients [2]. We present a case of bilateral stellate neuroretinitis due to pheochromocytoma.

## Patient and observation

**Figure 2:**
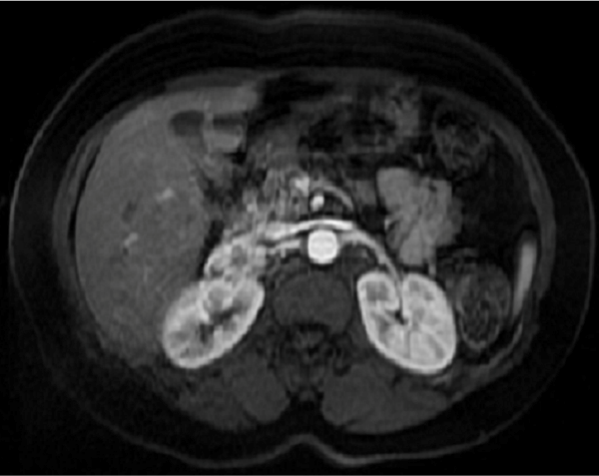
Magnetic resonance imaging identified a left suprarenal mass

**Figure 3:**
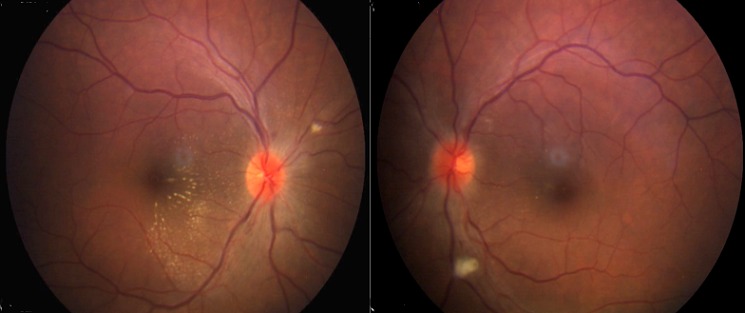
Fundus photograph 7 months after surgery

We report the case of a 33 year old woman patient admitted for a progressive bilateral visual loss since two weeks. Ophthalmologic examination revealed visual acuity of counting fingers in both eyes; and pupils were briskly reactive with no relative afferent papillary defect. Anterior segment examination in both eyes was unremarkable. Fundus examination showed bilateral stellate neuroretinitis. Physical examination revealed a malignant hypertension of 210/150mmHg. Magnetic resonance imaging identified a left suprarenal mass, whereas urinary catecholamine level was abnormally high which supported a diagnosis of pheochromocytoma (Figure 2). The patient underwent a laparoscopic left suprarenal adrenalectomy after successful control of blood pressure. histopathologic examination confirmed the diagnosis of pheochromocytoma. Postoperative evolution was uncomplicated. Antihypertensive treatment lasted only a few months. Visual acuity was restored and the retinal alterations disappeared 7 months after surgery (Figure 3).

## Discussion

Neuroretinitis is thought to be a result of an infectious or immune mediated process that may be precipitated by a number of bacterial, viral and parasitic agents [3]. The hallmark of neuroretinitis is optic disc edema with a macular star, which develops approximately 9-12 days after an onset and starts to disappear after 1 month, but can take 6-12 months for total resolution [4]. Neuroretinitis has been associated with infections agents such as cat-scratch disease, and noninfectious illnesses such as arteriovenous malformation, malignant hypertension, polyarteritis nodosa, inflammatory bowel disease, optic disc melanocytoma, pseudotumor cerebri, and sarcoidosis [5]. In severe systemic hypertension, particularly in young patients with minimal atherosclerosis, a fundus picture consisting of disc swelling and macular exudates often occurs. A macular star often forms, which, following treatment, regresses more gradually than the disc edema. The bilaterality of this condition usually differentiates it from idiopathic neuroretinitis; however, asymmetrical cases could create confusion. In addition, arteriolar constriction, cotton wool spots and retinal hemorrhages should suggest a diagnosis of hypertension. However, the diagnosis may be missed if accurate blood pressure measurement is not performed [6].

Blood pressure measurement is essential to exclude malignant hypertension. Blood glucose and erythrocyte sedimentation rate are necessary to help exclude diabetic retinopathy and arteritic optic neuropathy. A complete blood examination, including differential and morphology, may support the presence of infection and help exclude serious hematological problems that may potentiate venous occlusion. Syphilis serology should probably be performed, even in typical cases, because it can produce a similar fundus appearance, often during the early asymptomatic neurosyphilitic period. Additional tests may include fundus fluorescein angiography, cat scratch serology, Lyme disease serology, chest X-ray, angiotensin-converting enzyme level, lumbar puncture and neuroimaging [7]. Severe hypertensive retinopathy with optic neuropathy may be a consequence of malignant hypertension due to a pheochromocytoma. It is reversible after ablation of the tumor. Early diagnosis is of vital importance and relies on hormonal investigation and immunohistochemistry [8].

## Conclusion

This report underscores that a thorough history and meticulous clinical examination are irreplaceable, powerful diagnostic tools that can correctly direct the plan of management. Although neuroretinitis is the disease of varied etiology, and the extent of diagnostic workup should be determined by detailed history and examination. It is a potentially treatable condition with a favorable outcome [9].
